# Preschool Verbal and Nonverbal Ability Mediate the Association Between Socioeconomic Status and School Performance

**DOI:** 10.1111/cdev.13364

**Published:** 2020-03-23

**Authors:** Sophie von Stumm, Kaili Rimfeld, Philip S. Dale, Robert Plomin

**Affiliations:** ^1^ University of York York UK; ^2^ Institute of Psychiatry, Psychology & Neuroscience London UK; ^3^ University of New Mexico Albuquerque NM USA

## Abstract

We compared the extent to which the long‐term influence of family socioeconomic status (SES) on children's school performance from age 7 through 16 years was mediated by their preschool verbal and nonverbal ability. In 661 British children, who completed 17 researcher‐administered ability tests at age 4.5 years, SES correlated more strongly with verbal than nonverbal ability (.39 vs. .26). Verbal ability mediated about half of the association between SES and school performance at age 7, while nonverbal ability accounted for a third of the link. Only SES, but not verbal or nonverbal ability, was associated with changes in school performance from age 7 to 16. We found that SES‐related differences in school performance are only partly transmitted through children's preschool verbal abilities.

Children from impoverished backgrounds perform on average worse in school and achieve fewer educational qualifications than children from families of higher socioeconomic status (SES; Bradley & Corwyn, 2002; Schoon, Jones, Cheng, & Maughan, 2012; Sirin, 2005). This SES‐related discrepancy in school performance is already evident in the first school year and magnifies over the course of compulsory education (von Stumm, 2017). From a number of possible explanations for the observation that family SES is associated with school performance (Bradley & Corwyn, 2002; Tucker‐Drob, 2013), the linguistic socialization that children experience in early life in their respective family homes has received particular attention (e.g., Fernald, Marchman, & Weisleder, 2013; Hart & Risley, 1995; Hoff, 2013; Sperry, Sperry, & Miller, 2019). Specifically, high SES parents have been found to speak a greater number of words to their children, using larger and more complex vocabularies and referring to more abstract concepts, than parents of lower SES (Bernstein, 1975; Hart & Risley, 1995; Hoff, 2003). In turn, children from more advantaged family homes have been found to develop greater verbal ability themselves (Hart & Risley, 1995; Hoff, 2003), and they are more familiar, even before starting formal school, with the language patterns and linguistic codes that prevail in formal education settings and are expected by teachers (Heath, 1983; Lareau, 2003; van Bergen, van Zuijen, Bishop, & de Jong, 2017). By comparison, children from lower SES families experience less stimulating home language environments and acquire poorer verbal skills (Hart & Risley, 1995; Hoff, 2013; Pace, Luo, Hirsh‐Pasek, & Golinkoff, 2017), which are essential for participating in printed and oral academic language, for example in discussions and readings of scientific concepts and abstract physical and historical events. Home language environments and by extension, children's verbal abilities are thought to be modifiable (Morgan, Farkas, Hillemeier, Hammer, & Maczuga, 2015) and thus, they might make good targets for early interventions to improve children's long‐term verbal and academic outcomes (Dickinson, Golinkoff, & Hirsh‐Pasek, 2010), although the evidence for the effectiveness of such interventions is to date inconclusive (Bailey, Duncan, Odgers, & Yu, 2017; Haley, Hulme, Bowyer‐Crane, Snowling, & Fricke, 2017; Moreau, Macnamara, & Hambrick, 2018).

Many studies have shown that children's linguistic home environments differ systematically by SES, and that these differences are reflected in children's language skill and development (e.g., Hart & Risley, 1995; Heath, 1983; Hoff, 2003, 2013; Huttenlocher, Waterfall, Vasilyeva, Vevea, & Hedges, 2010). However, these studies have typically assessed small, nonrepresentative samples and did not include long‐term follow‐ups of the children as they progressed through school. By comparison, the predictive validity of children's academic and behavioral functioning at the time of kindergarten entry—typically referred to as “school readiness”—for their later academic achievement has been evidenced by several large‐scale studies (e.g., Duncan et al., 2007; Pace, Alper, Burchinal, Golinkoff, & Hirsh‐Pasek, 2019). That said, we identified only two well‐powered studies that focused on the association between children's preschool verbal ability and their later school performance. The first showed in a sample of 502 U.S. children that language skills in kindergarten, based on a comprehensive language test battery (i.e., picture vocabulary, oral vocabulary, sentence imitation, grammatical completion, and grammatical understanding), were positively associated with reading and mathematics achievement at the ages 7, 8, and 9 years (Durham, Farkas, Scheffner Hammer, Tomblin, & Catts, 2007). The second study demonstrated in a sample of 8,650 U.S. children that those with larger vocabularies at the age of 2 years performed better on measures of academic and behavioral functioning 3 years later (i.e., at kindergarten entry; Morgan et al., 2015). Although the authors of both studies considered numerous covariates and fitted relatively complex models to explore their data, three issues remain unaddressed. First, the studied samples were followed up during the early school years but the school performance at later stages up to the end of compulsory education was not evaluated. This later school performance is, however, of particular importance for children's long‐term educational trajectories, because the grades that children obtain at the end of compulsory schooling tend to inform their subsequent educational choices, such as opting for higher education over pursuing more applied vocational training and vice versa. Second, and perhaps related to the first issue of the limited follow‐up duration, the children's performance at the onset of school was not previously differentiated from the changes in their performance that occur over time. It is possible that the factors that influence children's differences in performance at the beginning of school differ from those that influence relative gains or losses in school performance over time (von Stumm, 2017). Finally, neither study allowed for a direct comparison between the effect of children's verbal and their other cognitive abilities on later school performance. This comparison is crucial, however, for substantiating the theory that SES‐related disadvantages are primarily transmitted via language socialization and thus, through children's verbal ability.

In the current study, we analyse data from a subsample from the Twins Early Development Study (TEDS), a longitudinal cohort study of twins born in England and Wales between 1994 and 1996, to address all three issues. Overall 750 TEDS families were visited at home when their twins were 4.5 years old (i.e., preschool) by trained research assistants, who administered a battery of 17 standardized verbal and nonverbal ability tests. Later the twins were followed‐up on school performance from age 7 to 16 years. We tested if family SES was more strongly associated with verbal than nonverbal ability in childhood, as well as the extent to which verbal and nonverbal ability mediated the long‐term influence of SES on children's differences in school performance at the start and throughout the duration of compulsory schooling.

## Method

### Sample

The study sample comes from the TEDS, a multivariate longitudinal study that recruited more than 11,000 twin pairs born in England and Wales in 1994 through 1996. The recruitment process and the sample are described in detail elsewhere (Haworth, Davis, & Plomin, 2013). The TEDS sample was at its inception representative of the U.K. population in comparison with census data and despite some attrition remains considerably representative (Kovas, Haworth, Dale, & Plomin, 2007).

A subsample of 750 TEDS families was visited at home by two trained research assistants between March 1998 and October 2001, when the twins were on average 4.5 years old, to administer an extensive cognitive test battery. This subsample is representative of the overall TEDS sample's range on all measures, although children at the lower end of ability were intentionally oversampled (Colledge et al., 2002; Viding et al., 2003). After excluding children who completed fewer than four cognitive tests, the maximum analysis sample included 743 unrelated individuals (i.e., one randomly selected twin per pair), for whom at least data on one data point relevant to the current analyses was available. For 661 individuals, verbal and nonverbal ability data were available at age 4.5.

### Measures

#### Verbal and NonVerbal Ability

Children completed eight language‐based tests and nine nonverbal cognitive ability measures, which were described in Colledge et al. (2002) and Viding et al. (2003) and are reviewed in detail in the Supplementary Material. The verbal tests included (a) story information, (b) grammar, (c) vocabulary, (d) word knowledge, (e) verbal fluency, (f) opposite analogies, (g) phonological awareness, and (h) articulation. For example, the word knowledge test asked children to explain words like “concert,” “factory,” and “towel,” while for verbal fluency, children were asked to name as many things as they could think of for verbs like “to eat.”

The nonverbal cognitive ability measures included (a) block building, (b) puzzle solving, (c) number questions, (d) tapping sequence, (e) draw and design, (f) draw a child, (g) numerical memory, (h) counting and sorting, and (i) conceptual grouping. For example, in number questions children were asked: “If you have four balloons and half of them broke, how many balloons will you have?”. For another example, children were asked to repeat a series of number sequences to assess their numerical memory.

#### School Performance

The twins’ teachers reported their scores in English and mathematics following the U.K. National Curriculum guidelines, which are formulated by the National Foundation for Educational Research (http://www.nfer.ac.uk/index.cfm) and the Qualifications and Curriculum Authority (http://www.qca.org.uk). At the twins' ages 7 through 14 years, teachers rated their achievement in English, including the categories “speaking,” “reading,” and “writing,” and Maths, including “use & applying,” “numbers,” and “shapes, spaces, and measures,” relative to “the national expected standard” of children of the same age on a 5‐point scale that ranged from 0 (*working to towards level 1*) and 1 (*level 1*), indicating achievement below the national expected standard, to 2 (*level 2*) that represented achievement at the expected standard, to 3 (*level 3*) and 4 (*level 4+*) that marked achievement above the national expected standard. The teacher ratings of English and mathematics correlated on average .70 from age 7 through to 14 with the test scores recorded in the U.K. National Pupil Database (NPD; Rimfeld et al., 2018). At age 16, parents reported the twins' scores in English and mathematics, which are subtests of the General Certificate of Secondary Education (GCSE) exam, a standardized examination taken at the end of compulsory schooling in the U.K. Parent‐reported GCSE scores for English correlated .98 and those for mathematics correlated .99 with the respective data recorded in the NPD. At each age, performance in English and mathematics were each represented by a single score.

#### Socioeconomic Status

Families' SES was assessed at the first contact, when the twins were 18 months old, with parents reporting their educational qualifications, their occupational positions, and the twins' mother's age at the birth of her first child. Educational qualifications ranged across 8 levels from “no formal education” to “postgraduate qualifications.” Occupational position was inferred from job title, employment status (i.e., do you have a job? Yes/no), job qualifications (i.e., do you need special qualifications for your job? Yes/no), and employment type (e.g., manager, self‐employed, foreman) in line with the standard occupational classification (Office of Population and Census Surveys, 1991). Age at first birth continues to have one of the strongest associations with women's SES (van Roode, Sharples, Dickson, & Paul, 2017). A composite measure of SES was calculated by taking a mean of standardized scores of mothers' and fathers' educational level, mothers' and father occupational status, and mothers' age on birth of the first child.

### Statistical Analyses

A series of analyses of variance (ANOVAs) tested for sex differences in the study variables, which we then corrected for sex and age differences using the regression method (i.e., saving standardized residuals, which were used in the subsequent analyses). A factor analysis of the 17 cognitive ability measures with varimax rotation suggested a two‐factor solution, with verbal tests loading more highly on one and nonverbal tests loading on the other factor, except for one nonverbal test that loaded higher on the verbal ability factor (i.e., numerical loaded .428 on verbal and .402 on nonverbal ability; cf. Colledge et al., 2002; Viding et al., 2003; see Table S1 for the test's correlations and  Table S2 for the factor loadings). The verbal ability factor accounted for 20.5% of the variance and the nonverbal ability factor for 19.1% of the variance in the ability tests. We saved regression factor scores, which represent orthogonal verbal ability and nonverbal ability factors, respectively (DiStefano, Zhu, & Mindrila, 2009), to use in the subsequent analyses.

After testing correlations across all study variables, we fitted a series of latent growth curve (LGC) models using the R package Lavaan (Rosseel, 2012). LGC models differentiate individual differences in the starting point of a trait, in our case school performance at age 7 (i.e., intercept factor), from individual differences in the rate of change that occurs over time in the same trait (i.e., slope factor), that is in the current study, the change in school performance that occurs from age 7 through 16 (i.e., performance gains).

LGC models were fitted separately for performance in English and mathematics, using the single score indicators from each assessment age (i.e., 7, 9, 10, 12, 14, and 16 years). In line with LGC modeling conventions, loadings for the intercept factor were set at 1, while factor loadings for the slope represented the time between assessment points in years (i.e., slope loadings were set at 0, 2, 3, 5, 7, and 9), thereby defining the starting point of the slope at age 7. We first tested one‐, two‐, and three‐factor LGC models of academic growth to identify which best represented the data. We then tested the predictive validity of early verbal and nonverbal factor scores, and in separate models that of SES as well, for school performance at age 7 (i.e., intercept factor) and of gains in school performance from age 7 through 16 years (i.e., slope factor).

To test if verbal and nonverbal ability mediated the path from SES to intercept and slope factors of English and mathematics performance, we applied the classical analysis model from Baron and Kenny (1986). In a first model, we regressed the growth factors for school performance (i.e., intercept and slope) onto SES, before regressing verbal and nonverbal ability onto SES in a second model. In a third step, we regressed SES and verbal and nonverbal ability onto school performance. If verbal and nonverbal ability fully mediated the effect of SES on school performance, the association between the latter should be zero in the third analysis step (Baron & Kenny, 1986). In case of a partial mediation, the association between SES and school performance would be significantly reduced by adding verbal and nonverbal ability factors to the model. Finally, we applied a Sobel's test to determine whether the mediation of the SES influence via verbal and nonverbal ability was significant. Because we tested mediations across four separate series of models (i.e., verbal and nonverbal ability on English and mathematics performance), we Bonferroni corrected the Sobel's test *p*‐values to .0125 (i.e., typical *p* of .05 divided by 4). All models used full information maximum likelihood estimation to deal with missing data, which were assumed to be missing at random.

## Results

Descriptive statistics for the overall sample and separately for males and females are shown in Tables S2 and S3. The ANOVAs revealed significant gender differences, with girls scoring higher in English at ages 7 and 9 and in nonverbal ability at age 4.5 than boys. However, on average, gender differences explained < 1% of the variance (Table S2).

**Figure 1:**
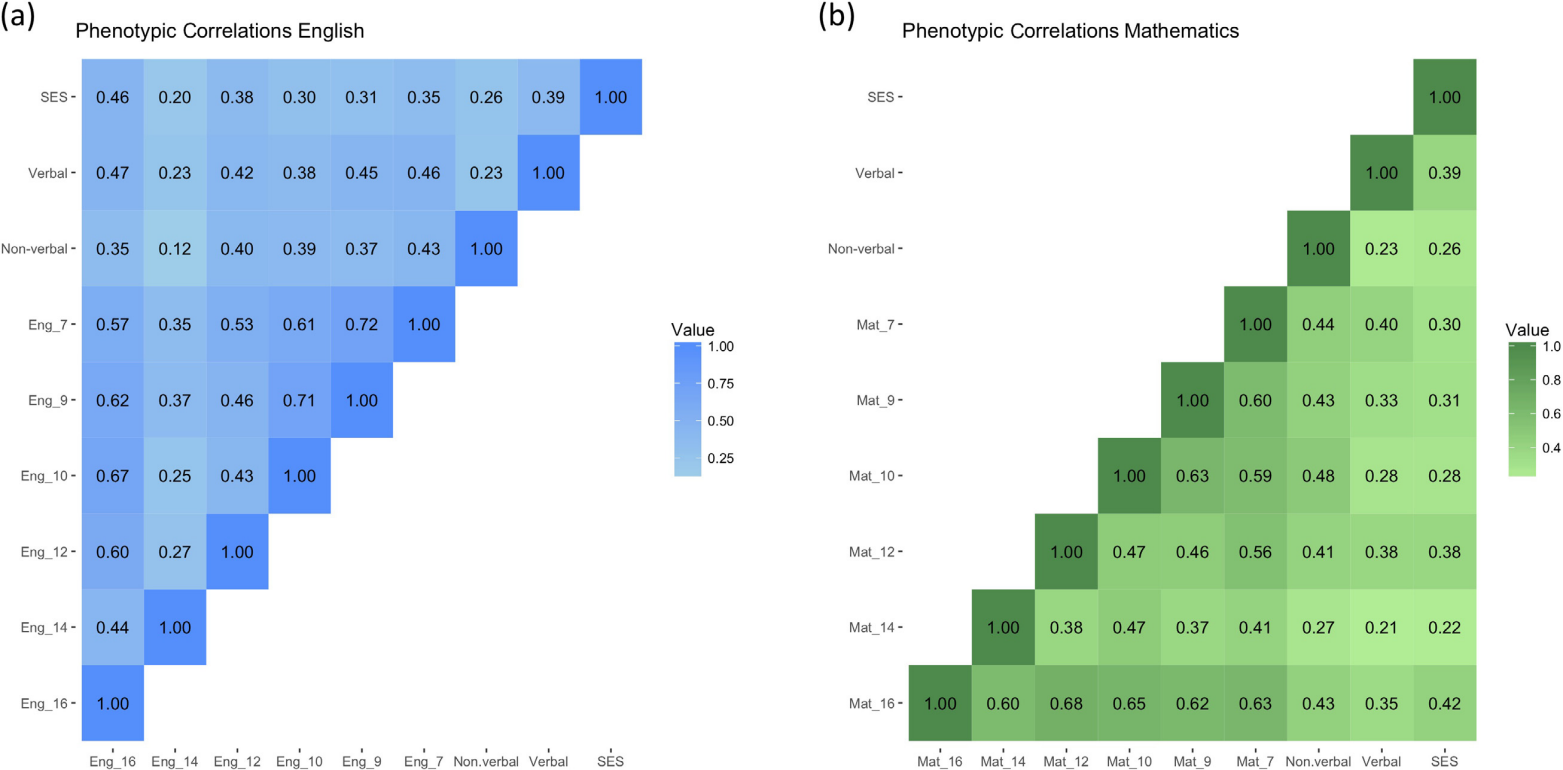
Correlations between socioeconomic status (SES), nonverbal and verbal ability and (a) English performance or (b) mathematics performance. [Color figure can be viewed at wileyonlinelibrary.com]

The full correlation matrix for the analysis sample is shown in Figure 1. SES correlated more strongly with 4.5 year verbal (.39) than nonverbal ability (.26), and this difference was significant (Fisher's *z* = 2.74, *p *= .003). SES was also positively correlated with later school performance, ranging from .20 to .46, with correlations between school performance and verbal ability and nonverbal ability showing a similar range. Overall, verbal ability was more strongly associated with performance in English, and nonverbal ability with mathematics. The highest correlations occurred among the school performance scores across years, reflecting their relatively high stability over time.

For English performance, a one‐factor growth model fitted significantly worse that a two‐factor solution (χ^2^_diff_ **= 13.75, *p *= .003), and a two‐factor model fitted worse than the three‐factor model (χ^2^_diff_ **= 14.27, *p *= .006). However, the third factor had zero between‐person variance; thus, we retained the two‐factor solution that differentiated intercept and slope factors for English performance (model fit: comparative fit index [CFI]* *= .944; root mean square error of approximation [RMSEA]* *= .060, CI 90% from .043 to .078). Similarly, for mathematics, the one‐factor growth model fitted significantly worse than a two‐factor solution (χ^2^_diff_ **= 13.51, *p *= .004), and a two‐factor model fitted worse than the three‐factor model (χ^2^_diff_ **= 11.88, *p *= .018). As with English, the third factor had zero between‐person variance, and we again retained a two‐factor solution (model fit: CFI* *= .969; RMSEA* *= .047, CI 90% from .029 to .066), which best represented the data. For English, the correlation between the intercept and slope factors was −.30; for mathematics, the corresponding correlation was .04.

Verbal and nonverbal ability were equally strong and significant predictors of the intercept of English performance (i.e., children's differences in English performance at age 7), and together they accounted for 46% of the variance in the intercept (Table 1). Verbal ability was not significantly associated with the slope (i.e., changes in English performance from age 7 through 16), but nonverbal ability was significantly, yet negatively related to the slope of English performance, accounting for 2.8% of the variance. We interpreted this association as a spurious finding, which did not replicate in sensitivity analyses in a sample including the other randomly selected twin per pair. In contrast with the results for performance in English, nonverbal ability was significantly more predictive of mathematics at age 7 (i.e., intercept) than was verbal ability (i.e., betas of .50 and .38; Fisher's *z *= 2.75, *p *= .003), accounting together for 49% of the variance (Table 1). Neither verbal nor non‐verbal ability were significantly associated with changes in mathematics over time (i.e., slope).

**Table 1:** Parameter Estimates for Socioeconomic Status (SES) and Verbal and Nonverbal Ability as Predictors of Latent Growth Factors (Intercept and Slope) of School Performance

	β_i_	*SE*_i_	*p*_i_	*R* ^2^_i_	β_s_	*SE*_s_	*p*_s_	*R* ^2^_s_
English performance								
Model 1								
Verbal ability	.436	.035	< .001	.463	.053	.005	.515	.028
Nonverbal ability	.431	.036	< .001		−.172	.005	.039	
Model 2								
Verbal ability	.402	.038	< .001	.473	−.058	.005	.504	.123
Nonverbal ability	.413	.037	< .001		−.245	.005	.004	
SES	.101	.038	.031		.335	.005	< .001	
Mathematics performance								
Model 1								
Verbal ability	.382	.035	< .001	.486	−.069	.005	.505	.005
Nonverbal ability	.502	.035	< .001		.003	.005	.980	
Model 2								
Verbal ability	.344	.037	.049	.492	−.203	.005	.057	.155
Nonverbal ability	.486	.036	< .001		−.083	.005	.426	
SES	.097	.037	< .001		.432	.005	< .001	

Note

i = intercept; s = slope; β = standardized coefficient; *SE* = standard error; *p* = *p*‐value; *R* ^2^ = adjusted *R* ^2^.

SES was significantly associated with the intercept in both English (β* *= .39, *SE *= .04, *p* < .001) and mathematics performance (β* *= .37, *SE *= .04, *p* < .001; difference not significant, Fisher's *z *= 0.07, *p *= .472). SES was also strongly associated with the slopes, that is, with performance gains from age 7 through 16 in English (β* *= .25, *SE *= .01, *p *= .003), and even significantly more so for mathematics (β* *= .34, *SE *= .00, *p *= .001; Fisher's *z *= 1.87, *p *= .031). In a model with SES as a predictor beyond verbal and nonverbal ability (Table 1), SES continued to be comparably associated with the intercepts in English and mathematics (Fisher's *z *= 0.07, *p *= .472) and more strongly with the slope in mathematics than in English performance (Fisher's *z *= 2.00; *p *= .023). We fitted an additional set of LGC models that used mothers' highest level of education rather than the SES index variable to operationalize family background. The results did not differ across both operationalizations of family background.

**Figure 2:**
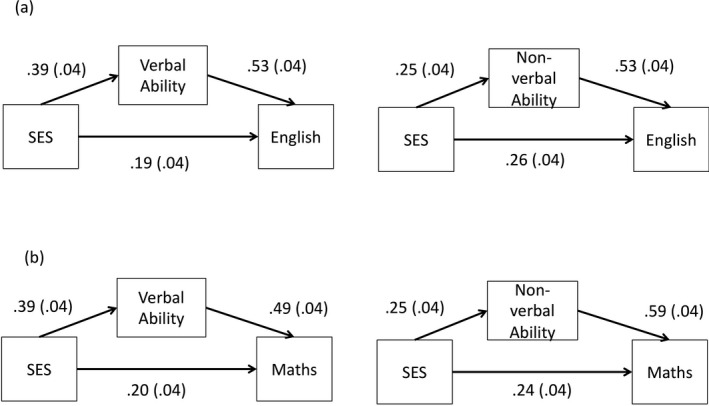
Mediation by verbal and nonverbal ability at age 4.5 years of the association between socioeconomic status and performance in English and mathematics. *Note*. Paths weights are standardized estimates. Standard Errors are shown in parentheses.

The mediation analyses showed that both verbal and nonverbal ability partially mediated the association between SES and school performance (verbal ability: Sobel's_English_ **= 7.37, *SE *= .02, *p* < .001; Sobel's_Mathematics_ **= 6.80, *SE *= .02, *p* < .001; nonverbal ability: Sobel's_English_ **= 5.46, *SE *= .02, *p* < .001; Sobel's_Mathematics_ **= 5.46, *SE *= .02, *p* < .001). These results are illustrated in Figure 2, with the direct path from SES to English performance of .39 reducing to .19 and .26 after adjusting for verbal and nonverbal ability (Figure 2a), respectively, and that to mathematics performance lowering from .37 to .20 and .24 (Figure 2b).

**Figure 3:**
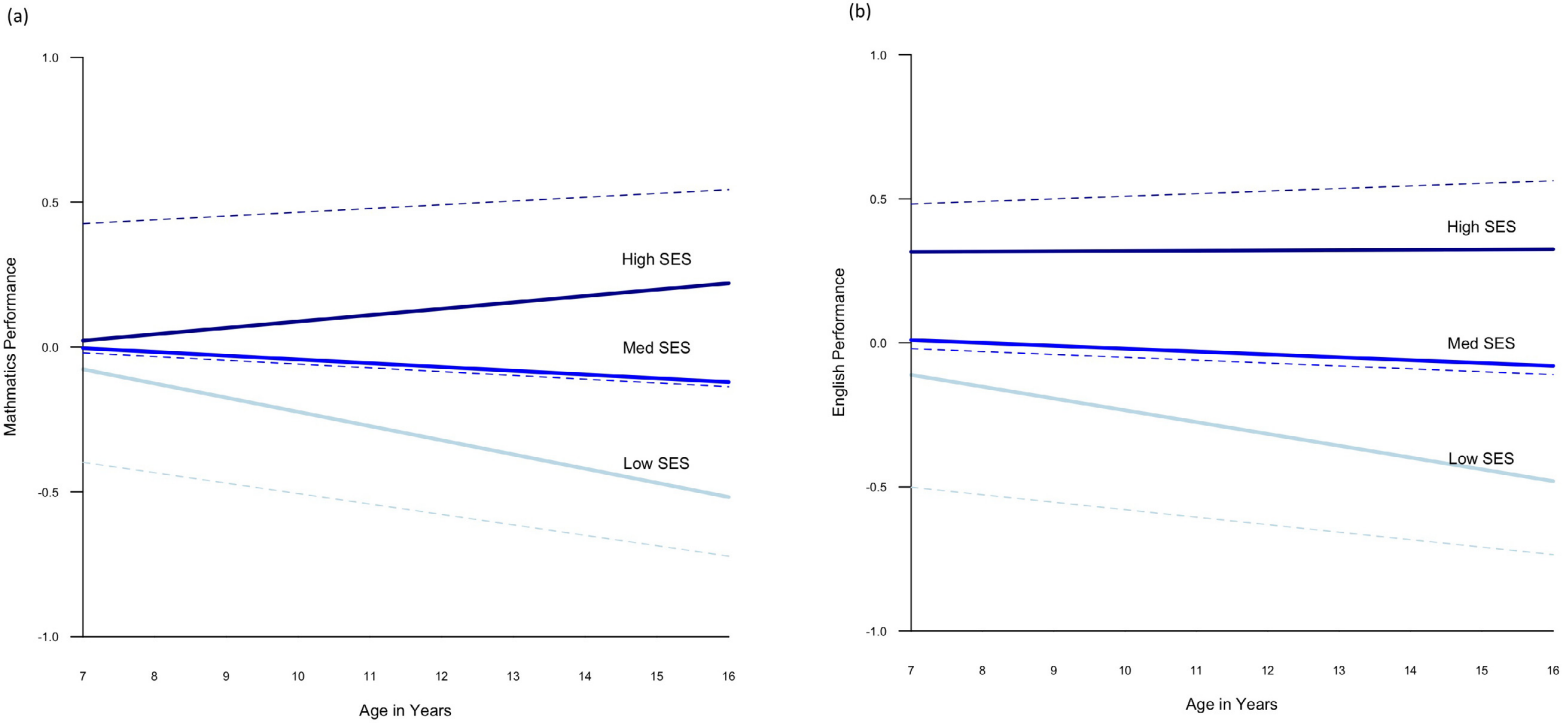
Performance trajectories for children from low, medium, and high socioeconomic status (SES) backgrounds, with and without adjusting for verbal and nonverbal ability. Trajectories in mathematics are shown in (a); trajectories in English are shown in (b). *Note*. Low SES includes children, whose family SES is 1 *SD* below the mean (*N* = 170); medium SES includes children, whose family SES is between −1 and +1 *SD* (*N* = 488); and high SES includes children, whose family SES is 1 *SD* above the mean (*N* = 177). The dotted lines show education trajectories for each SES group without adjusting for verbal and nonverbal ability; the straight lines represent trajectories after adjusting for differences in verbal and nonverbal ability. [Color figure can be viewed at wileyonlinelibrary.com]

Thus, verbal and nonverbal ability accounted for 50% and 32% of the association between SES and English performance at age 7, and for 46% and 35% of the association between SES and mathematics performance at age 7. Because verbal and nonverbal ability were not significant predictors of changes in school performance from age 7 through 16 (i.e., slope), they did not meet the criteria of mediating variables for later school performance (Baron & Kenny, 1986). Figure 3 illustrates that verbal and nonverbal ability partly account for the SES‐related differences in children's school performance at age 7 (i.e., intercept). By comparison, the slopes are only marginally altered after adding verbal and nonverbal ability to the model, reflecting that neither verbal nor nonverbal ability were associated with changes in school performance in the current analyses.

## Discussion

Children from low SES families have been shown to experience impoverished language environments, which are thought to impair verbal development (e.g., Bernstein, 1975; Fernald et al., 2013; Hart & Risley, 1995; Heath, 1983) and their later school performance (Hoff, 2013; Pace et al., 2017). Based on our analyses of a subsample of a U.K. representative cohort study, we add to the existing empirical evidence in three ways. First and in line with previous research, we observed that SES correlated more strongly with children's verbal than their nonverbal ability at age 4.5 years (e.g., Hoff, 2003; Huttenlocher et al., 2010), which suggests that children’s family background is to a greater extent reflected by verbal than nonverbal abilities (Hart & Risley, 1995; Heath, 1983), at least in early life. Second, we found that both verbal and nonverbal ability were significant mediators of the association between SES and children's differences in school performance at age 7, but verbal ability mediated the relation to a greater extent than nonverbal ability. Specifically, verbal ability accounted for 50% and 46% of the effect of SES on English and mathematics performance, while nonverbal ability accounted for 32% and 35% of these effects. On the one hand, this finding supports the hypothesis that SES‐related differences in school performance are transmitted to a greater extent by children's verbal abilities than their nonverbal abilities (Durham et al., 2007; Morgan et al., 2015). On the other hand, we note that the effect size of nonverbal ability as mediator was—independent of verbal ability—considerable, suggesting that the full range of children’s cognitive abilities is relevant for understanding the influence of SES on to school performance (Tucker‐Drob, 2013; von Stumm, 2017; von Stumm & Plomin, 2015).

Third, neither verbal nor nonverbal ability at age 4.5 years were meaningfully associated with changes in children’s relative level of school performance over time. Preschool cognitive abilities were related to those differences in children's school performance that are stable over the course of compulsory schooling, suggesting that they exert a continuous influence on school performance. However, preschool cognitive abilities were less relevant for children's relative performance gains that occurred later. By comparison, SES was not only a significant predictor for performance differences that were stable from the start of school but also influenced children’s changes in school performance over time, accounting for up to 12% of the variance in the latter: Children from more advantaged family homes improved to a greater extent in school performance over time relative to children from low SES backgrounds. Finding that the long‐term influence of SES on change in school performance was independent of early life verbal and nonverbal ability implies that other factors must mediate the influence of SES at the later stages of schooling, for example, the overall quality of the school, and differential access to educational support systems when needed, such as tutoring. Given that verbal and nonverbal ability only partly mediated the effect of SES on school performance at age 7, it is of course possible that these other factors, which we did not analyse in the current study, are also relevant mediators at the start of school. We note here, too, that the observed associations between SES, cognitive abilities, and school performance are at least partially mediated by genetic factors (Krapohl & Plomin, 2015).

### Limitations and Future Directions

Our study has some notable strength, including a comparatively large sample for whom high‐quality cognitive test data in early life, as well as longitudinal assessments of school performance, were available. Our study also has two key limitations. First, we focused here on early life cognitive abilities as mediators for the association between SES and school performance, but other potentially important factors, for example parent's attitudes to school and education or their own ability levels, were not considered (Tucker‐Drob, 2013). Ascertaining the additional mediators of the influence of SES on children's school performance will be crucial for implementing successful interventions that improve the equality of children's life chances and thus, should be a priority for future research. Second, our analyses did not explore the extent to which genetic factors may explain the observed associations between family background, children’s early life verbal and nonverbal ability and their school performance (Scarr & McCartney, 1983). As a result, mediation in the current study concerns mainly the prediction of later outcomes, which is highly relevant for the planning and application of interventions, but does not imply causality.

### Conclusions

We have shown here that differences in family SES are more strongly reflected by children's preschool verbal rather than by their nonverbal ability, both of which were comparable predictors of later school achievement. Because verbal ability mediated the association between SES and school performance to a greater extent than nonverbal ability, the current findings suggest that language plays a special role for transmitting SES‐related disadvantages, at least for those differences in children's school performance that are stable from the early school years onwards. However, our findings also raise doubts that interventions that solely target preschool verbal ability will eliminate the pervasive long‐term influence of family background on school performance.

## Supporting information


**Table S1. **Correlations for Cognitive Ability Tests at Age 4.5
**Table S2. **Factor Loadings for Cognitive Ability Tests at Age 4.5 After Varimax Rotation
**Table S3. **Descriptive Statistics for Verbal and Nonverbal Ability at Age 4.5 and for School Performance From Age 7 to 16
**Table S4. **Descriptive Statistics for Males and Females Separately for Verbal and Nonverbal Ability at Age 4.5 and for School Performance From age 7 to 16
**Appendix S1. **Detailed Descriptions of (a) Verbal Ability Tests and (b) Nonverbal Ability Tests Administered at Age 4.5
