# Supplementary material for: Preschool Verbal and Nonverbal Ability Mediate the Association Between Socioeconomic Status and School Performance
Source: Child Dev. 2020 Mar 23;91(3):705–14. doi: 10.1111/cdev.13364 (PMC7317529; doi:10.1111/cdev.13364)
Supplement: Supplementary file 1 — Table S1. Correlations for Cognitive Ability Tests at Age 4.5 Table S2. Factor Loadings for Cognitive Ability Tests at Age 4.5 After Varimax Rotation Table S3. Descriptive Statistics for Verbal and Nonverbal Ability at Age 4.5 and for School Performance From Age 7 to 16 Table S4. Descriptive Statistics for Males and Females Separately for Verbal and Nonverbal Ability at Age 4.5 and for School Performance From age 7 to 16 Appendix S1. Detailed Descriptions of (a) Verbal Ability Tests and (b) Nonverbal Ability Tests Administered at Age 4.5 [file CDEV-91-705-s001.docx]

**SUPPLEMENTARY MATERIALS**

**Pre-school verbal and non-verbal ability mediate the association between socioeconomic status and school performance**

**Supplementary tables**

**Table S1** Correlations for cognitive ability tests at age 4.5

Table S2. **.** Factor loadings for cognitive ability tests at age 4.5 after varimax rotation

**Table S3.** Descriptive statistics for verbal and non-verbal ability at age 4.5 and for school performance from age 7-16

**Table S4.** Descriptive statistics for males and females separately for verbal and non-verbal ability at age 4.5 and for school performance from age 7-16.

**Supplementary methods.** Detailed descriptions of (a) verbal ability tests and (b) non-verbal ability tests administered at age 4.5

**Table S1.** Correlations for cognitive ability tests at age 4.5

|  | **1** | **2** | **3** | **4** | **5** | **6** | **7** | **8** | **9** | **10** | **11** | **12** | **13** | **14** | **15** | **16** | **17** |
| --- | --- | --- | --- | --- | --- | --- | --- | --- | --- | --- | --- | --- | --- | --- | --- | --- | --- |
| Bus story information total score (1) | 1 |  |  |  |  |  |  |  |  |  |  |  |  |  |  |  |  |
| Action pictures grammar total score (2) | .569 | 1 |  |  |  |  |  |  |  |  |  |  |  |  |  |  |  |
| BAS (British ability scale) total score (3) | .448 | .467 | 1 |  |  |  |  |  |  |  |  |  |  |  |  |  |  |
| Word knowledge total score (4) | .580 | .510 | .465 | 1 |  |  |  |  |  |  |  |  |  |  |  |  |  |
| Verbal Fluency total score (5) | .504 | .487 | .480 | .514 | 1 |  |  |  |  |  |  |  |  |  |  |  |  |
| Opposite analogies total score (6) | .464 | .494 | .507 | .492 | .463 | 1 |  |  |  |  |  |  |  |  |  |  |  |
| Phonological awareness total score (7) | .380 | .376 | .371 | .396 | .369 | .384 | 1 |  |  |  |  |  |  |  |  |  |  |
| Articulation total score (8) | .316 | .411 | .471 | .335 | .373 | .387 | .319 | 1 |  |  |  |  |  |  |  |  |  |
| Block building total score (9) | .240 | .217 | .344 | .300 | .275 | .270 | .270 | .265 | 1 |  |  |  |  |  |  |  |  |
| Puzzle solving total score (10) | .314 | .268 | .297 | .297 | .296 | .329 | .238 | .252 | .342 | 1 |  |  |  |  |  |  |  |
| Number questions total score (11) | .362 | .388 | .446 | .447 | .423 | .476 | .360 | .313 | .301 | .230 | 1 |  |  |  |  |  |  |
| Tapping sequence total score (12) | .285 | .258 | .272 | .220 | .279 | .297 | .239 | .303 | .274 | .284 | .257 | 1 |  |  |  |  |  |
| Draw a design total score (13) | .267 | .309 | .330 | .313 | .348 | .324 | .333 | .317 | .327 | .382 | .363 | .338 | 1 |  |  |  |  |
| Draw a child total score (14) | .210 | .279 | .280 | .272 | .273 | .247 | .237 | .250 | .269 | .262 | .258 | .228 | .364 | 1 |  |  |  |
| Numerical memory total score (15) | .401 | .348 | .375 | .415 | .394 | .434 | .371 | .309 | .242 | .256 | .399 | .343 | .332 | .229 | 1 |  |  |
| Counting and sorting total score (16) | .354 | .430 | .491 | .412 | .441 | .446 | .374 | .424 | .339 | .370 | .454 | .396 | .407 | .318 | .404 | 1 |  |
| Conceptual grouping total score (17) | .341 | .364 | .462 | .416 | .374 | .399 | .381 | .305 | .321 | .312 | .384 | .289 | .320 | .272 | .357 | .496 | 1 |

| **Table S2.** Factor loadings for cognitive ability tests at age 4.5 after varimax rotation | | |
| --- | --- | --- |
|  | Factor | |
|  | 1 | 2 |
| Bus story information total score | **0.776** | 0.124 |
| Action pictures grammar total score | **0.650** | 0.260 |
| BAS (British ability scale) total score | **0.489** | 0.474 |
| Word knowledge total score | **0.683** | 0.261 |
| Verbal Fluency total score | **0.581** | 0.362 |
| Opposite analogies total score | **0.561** | 0.405 |
| Phonological awareness total score | **0.419** | 0.392 |
| Articulation total score | 0.356 | **0.451** |
| Block building total score | 0.191 | **0.479** |
| Puzzle solving total score | 0.225 | **0.455** |
| Number questions total score | 0.441 | **0.453** |
| Tapping sequence total score | 0.185 | **0.477** |
| Draw a design total score | 0.200 | **0.574** |
| Draw a child total score | 0.163 | **0.406** |
| Numerical memory total score | **0.428** | 0.402 |
| Counting and sorting total score | 0.342 | **0.654** |
| Conceptual grouping total score | 0.334 | **0.515** |

*Note*: Extraction method: Generalized Least Squares; Rotation converged in 3 iterations.

**Table S3.** Descriptive statistics for verbal and non-verbal ability at age 4.5 and for school performance from age 7 to 16 years

|  | n | mean | sd | min | max | skew | kurtosis | F Sex differences | R^2^ |
| --- | --- | --- | --- | --- | --- | --- | --- | --- | --- |
| Verbal ability age 4.5 | 661 | 0 | 0.88 | -2.27 | 2.88 | 0.37 | -0.11 | 0.59 | <0.01 |
| Non-verbal ability age 4.5 | 661 | 0 | 0.86 | -2.52 | 2.7 | -0.02 | 0.02 | 27.68** | 0.04 |
| English age 7 | 483 | -0.21 | 1.11 | -3.73 | 2.74 | -0.56 | 0.69 | 18.22** | 0.04 |
| Mathematics age 7 | 481 | -0.15 | 1.07 | -3.69 | 3.23 | -0.37 | 0.8 | 0.53 | <0.01 |
| English age 9 | 213 | -0.19 | 1.06 | -3.08 | 2.52 | -0.39 | 0.26 | 10.31** | 0.05 |
| Mathematics age 9 | 214 | -0.1 | 1.05 | -2.94 | 2.48 | -0.21 | -0.11 | 0.00 | <0.01 |
| English age 10 | 228 | -0.14 | 1.07 | -3.34 | 2.26 | -0.52 | 0.44 | 1.90 | 0.01 |
| Mathematics age 10 | 225 | -0.12 | 1.01 | -3.20 | 2.20 | -0.48 | 0.53 | 1.70 | 0.01 |
| English age 12 | 339 | -0.03 | 0.90 | -2.57 | 2.90 | 0.18 | 0.45 | 1.17 | 0.01 |
| Mathematics age 12 | 333 | 0.03 | 0.90 | -2.37 | 2.88 | 0.04 | 0.04 | 0.92 | <0.01 |
| English age 14 | 266 | 5.56 | 1.52 | -1.00 | 9.00 | -2.25 | 7.92 | 2.42 | 0.01 |
| Mathematics age 14 | 267 | 6.16 | 1.74 | -1.00 | 9.00 | -1.97 | 6.12 | 0.07 | <0.01 |
| English age 16 | 497 | 8.62 | 1.26 | 5.00 | 11.00 | -0.11 | -0.31 | 5.66* | 0.01 |
| Mathematics age 16 | 501 | 8.64 | 1.52 | 4.00 | 11.00 | -0.43 | -0.09 | 1.80 | <0.01 |
| SES^♮^ | 697 | -0.02 | 1.04 | -2.47 | 2.50 | 0.13 | -0.74 | 0.59 | <0.01 |

Note: N= one randomly selected twin per pair (sample 1); F= F statistics from ANOVA; p<0.001; *<0.05.

**Table S4.** Descriptive statistics for males and females separately for verbal and non-verbal ability at age 4.5 and for school performance from age 7 to 16 years.

| **Male** |  |  |  |  |  |  |  |
| --- | --- | --- | --- | --- | --- | --- | --- |
|  | n | mean | sd | min | max | skew | kurtosis |
| Verbal ability age 4.5 | 350 | 0.02 | 0.84 | -1.89 | 2.88 | 0.34 | -0.11 |
| Non-verbal ability age 4.5 | 350 | -0.16 | 0.83 | -2.46 | 2.7 | 0.15 | 0.17 |
| English age 7 | 258 | -0.41 | 1.15 | -3.73 | 2.14 | -0.55 | 0.32 |
| Maths age 7 | 258 | -0.18 | 1.16 | -3.69 | 1.5 | -0.59 | 0.63 |
| English age 9 | 109 | -0.42 | 1.08 | -3.08 | 1.53 | -0.34 | -0.03 |
| Maths age 9 | 109 | -0.10 | 1.08 | -2.94 | 1.51 | -0.25 | -0.35 |
| English age 10 | 114 | -0.24 | 1.12 | -3.34 | 2.26 | -0.50 | 0.51 |
| Maths age 10 | 112 | -0.03 | 1.02 | -3.20 | 2.2 | -0.63 | 1.02 |
| English age 12 | 178 | -0.08 | 0.93 | -2.20 | 2.9 | 0.54 | 0.97 |
| Maths age 12 | 174 | 0.07 | 0.90 | -2.37 | 2.88 | 0.28 | 0.27 |
| English age 14 | 137 | 5.42 | 1.40 | -1.00 | 8.00 | -1.95 | 7.66 |
| Maths age 14 | 138 | 6.19 | 1.63 | -1.00 | 9.00 | -1.72 | 5.98 |
| English age 16 | 261 | 8.49 | 1.28 | 5.00 | 11.00 | 0.03 | -0.42 |
| Maths age 16 | 264 | 8.73 | 1.46 | 4.00 | 11.00 | -0.37 | -0.14 |
| SES | 397 | 0 | 1.03 | -2.47 | 2.49 | 0.08 | -0.72 |
|  |  |  |  |  |  |  |  |
| **Female** |  |  |  |  |  |  |  |
| Verbal ability age 4.5 | 311 | -0.03 | 0.93 | -2.27 | 2.81 | 0.41 | -0.17 |
| Non-verbal ability age 4.5 | 311 | 0.18 | 0.85 | -2.52 | 2.39 | -0.24 | 0.17 |
| English age 7 | 225 | 0.02 | 1.02 | -3.73 | 2.74 | -0.47 | 1.07 |
| Maths age 7 | 223 | -0.11 | 0.96 | -2.55 | 3.23 | 0.14 | 0.57 |
| English age 9 | 104 | 0.04 | 0.99 | -3.08 | 2.52 | -0.38 | 0.57 |
| Maths age 9 | 105 | -0.11 | 1.01 | -2.94 | 2.48 | -0.17 | 0.13 |
| English age 10 | 114 | -0.04 | 1.02 | -3.34 | 2.26 | -0.49 | 0.17 |
| Maths age 10 | 113 | -0.21 | 1.00 | -3.20 | 2.20 | -0.32 | 0.06 |
| English age 12 | 161 | 0.02 | 0.87 | -2.57 | 2.18 | -0.28 | -0.14 |
| Maths age 12 | 159 | -0.02 | 0.90 | -2.37 | 1.63 | -0.24 | -0.39 |
| English age 14 | 129 | 5.71 | 1.64 | -1.00 | 9.00 | -2.51 | 8.21 |
| Maths age 14 | 129 | 6.13 | 1.86 | -1.00 | 9.00 | -2.09 | 5.80 |
| English age 16 | 236 | 8.76 | 1.23 | 5.00 | 11.00 | -0.25 | -0.08 |
| Maths age 16 | 237 | 8.54 | 1.59 | 4.00 | 11.00 | -0.45 | -0.14 |
| SES | 318 | -0.04 | 1.05 | -2.46 | 2.50 | 0.19 | -0.75 |

**Supplementary methods**

1. **Verbal ability**

Intelligence at age 4.5 was measured in-person. Verbal score was created from 8 language based tests, including Bus story information, action pictures grammar score, BAS total score, word knowledge, verbal fluency, opposite analogies, phonological awareness, and articulation, which are described below.

A. Bus story information.

Bus story is a verbal comprehension task (Renfrew, 1997). For example, the researcher said: **“I’m going to tell you a story about this bus** [point]**, then, when I’m finished, I want *you* to tell *me* the story about this bus.”**

1. Once upon a time there was a very naughty bus.
   While his driver was trying to mend him, the bus decided to run away.
2. He ran along the road beside a train.
   They made funny faces at each other and raced each other.
   But the bus had to go on the alone, because the rain went into a tunnel. He hurried into the city where he met a policeman who blew his whistle and shouted “stop, bus”.
3. But the naughty bus paid no attention and ran on into the country. He said, “I’m tired of going on the road”. So he jumped over a fence. He met a cow who said, “Moo, I can’t believe my eyes”.
4. The bus raced down the hill. As soon as he saw there was water at the bottom, he tried to stop. But he didn’t know how to put on his brakes. So he fell in the pond with a splash and stuck in the mud. When the driver found where the bus was, he telephoned for a crane to pull him out and put him back on the road.

The child responses were recorded on the tape recorder**. Researcher**: “**Now *you* tell *me* the story. Once upon a time there was a...”** [turn pages as child tells story]
**And then...
So... “**

The descriptions given by the children were rated in terms of information, sentence length and subordinate clauses. For example: **Bold type** = 2 points (1 point if half response is correct), Normal type = 1 point .

bus naughty; driver mending/fixing; bus **ran away/drove off; train in tunnel** bus **alone;** policeman blew whistle / **said Stop;**

B. Action pictures grammar score

Children were shown action pictures and were asked to describe the pictures. Their responses were recorded, and a score was given for each picture in terms on information given and grammar used (Renfrew, 1988).

C. BAS (British Ability Scales) total score

Verbal Comprehension test (Elliot, Smith, & McCulloch, 1996). For example, the researcher: “**What is this? Yes, it’s a teddy bear. Have a good look at him**”.

1)  Show me teddy’s legs

2)  Show me teddy’s mouth

3)  Show me teddy’s eyes

Total of 27 questions were asked, all correct responses were given the score of 1, incorrect responses were given a score of 0. Correct answers were added up for the total BAS score.

D. Word knowledge

The word knowledge test consisted of 2 parts (McCarthy, 1972). Part 1 tested picture vocabulary where children were shown pictures and they had to name the objects on the pictures. For example, clock, sailboat. If children scored less than 6 (out of 9) then they discontinued after part 1.

Part 2 tested oral vocabulary. Children were asked to explain the words given, for example, towel, coat, tool. Responses were coded 0 if no correct answer was given to 2 were perfect explanation was given. Maximum score was 20 (perfect explanation to 10 words).

E. Verbal fluency

Children were given 20 seconds to name as many things as possible in every category (things to eat, animals, things to wear, things to ride). Maximum score was 36 (McCarthy, 1972).

F. Opposite analogies

Children had to complete the sentences (McCarthy, 1972). For example: the sun is hot, the ice is ___; I throw the ball up, and then it comes ___. Children were presented with 9 sentences to complete, each correct answer gave them 1 point (maximum score 9).

G. Phonological awareness

Puppet presented children with a word and children had to choose a card with a word that rhymed with the target word. If child selects wrong item say “**No!**” with puppet shaking head, and have a puppet indicate which card is the right one, saying, for example, “**Lynn likes the bin because bin sounds like Lynn**”. Each correct answer was scored as 1. Maximum score was 12 (Bird, Bishop, & Freeman, 1995).

H. Articulation

Children were presented with 35 words and their articulation was assessed using the Non-Word repetition test and Goldman Fristoe test, a composite score of these 2 tests was used for the measure of articulation (Gathercole, Willis, Baddeley, & Emslie, 1994; Goldman & Fristoe, 1986).

1. **Non-verbal ability**

Non-verbal score was created from 9 non-verbal test scores, including block building, puzzle solving, number questions, tapping sequence, draw and design, draw a child, numerical memory, counting and sorting, and conceptual grouping. All the tests were based on McCarthy Scales of Children’s Abilities (McCarthy, 1972).

A. Block building

Children were asked to build a tower; a chair; a building; a house from the blocks, they were given 2 trials to do it and scored from 0-3 for each item. Children discontinued after failure on both trials after 2 consecutive items.

B. Puzzle solving

Children were asked to solve puzzles. They were given 30 seconds to solve first three puzzles. Children discontinued after 3 consecutive failures. Then time increased for 4^th^ puzzle to 60 seconds, 5^th^ puzzle to 90 seconds and to 120 seconds for the 6^th^ puzzle. Bonus points were given to items 4-6 if child completed the puzzle quickly, but only if the puzzle was completed perfectly.

C. Number questions

Children were asked 12 number questions. For example: how many ears do you have; if you have 9 pennies and you lose 3 of them, how many will you have left? Children discontinued after 4 consecutive failures.

D. Tapping sequence

Children were asked to repeat a tapping sequence. Children only continued with the test if the first sequence was correct and were given 3 trials to do it. Children discontinued after 2 consecutive failures. Children were presented with 8 sequences.

E. Draw and design

Children were asked to copy a design (draw it) starting from circle and vertical or horizontal lines to more complex figures. Children had to draw 9 designs and discontinued after 3 consecutive failures.

F. Draw a child

Children were asked to draw a child. The maximum score given was 20. Points were given based on different features included in the drawing: head, hair, eyes, nose, mouth, neck, trunk, arms and hands, attachment of arms, legs and feet.

G. Numerical memory

Children had to repeat series of number sequences, starting from 2 numbers and finishing with 7 numbers. Six trials included forward series and five trials backward series. Children were given 2 trials per sequence. Discontinue rule applied in forward series after failure on *both* trials of any item. If child earned 3 or more points in forward trials, then backward trials were presented and discontinued after failure on any item.

H. Counting and sorting

Children were asked to count and sort blocks. A series of 9 questions were presented.

I. Conceptual grouping

Children were asked to group blocks. For example based on size (little, big), based on color (red, yellow, blue) or shape (square, round) as the test progressed groupings were more difficult combining features (large, blue, square). Children were given 9 tasks for grouping and they discontinued after 9 consecutive failures.

**REFERENCES**

Bird, J., Bishop, D. V. M., & Freeman, N. H. (1995). Phonological Awareness and Literacy Development in Children with Expressive Phonological Impairments. *Journal of Speech and Hearing Research*, *38*, 446–462.

Elliot, C. ., Smith, P., & McCulloch, K. (1996). *Verbal Comprehension Scale from British Ability Scales (2nd ed)*. Windsor: NFER-Nelson.

Gathercole, S. E., Willis, C. S., Baddeley, A. D., & Emslie, H. (1994). The Children’s Test of Nonword Repetition: A Test of Phonological Working Memory. *Memory*, *2*(2), 103–127. http://doi.org/10.1080/09658219408258940

Goldman, R., & Fristoe, M. (1986). *Goldman Fristoe Test of Articulation*. Cicle Pines, MN, American Guidance Service.

McCarthy, D. (1972). *McCarthy Scales of Children’s Abilities*. New York: The Psychological Corporation.

Renfrew, C. . (1988). *Action Picture Test (4th ed.)*. Bicester: Winslow Press Ltd.

Renfrew, C. . (1997). *Bus Story Test – a test of narrative speech (4th ed)*. Bicester: Winsslow Press Ltd.
